# P-1749. Implementation of an Automatic 36-hour Stop Order on Empiric Meropenem Usage

**DOI:** 10.1093/ofid/ofae631.1912

**Published:** 2025-01-29

**Authors:** Connor Parker, Jack G Schneider, LaKeisha Boyd, Michelle L Kussin

**Affiliations:** Indiana University School of Medicine, Indianapolis, Indiana; Indiana University School of Medicine, Indianapolis, Indiana; Indiana University, Indianapolis, Indiana; IU Health, Indianapolis, Indiana

## Abstract

**Background:**

Implementation of automatic stop orders (ASOs) for empiric antimicrobials have reduced antimicrobial use without negatively impacting patient outcomes. Given a recent increase in empiric meropenem use at our tertiary referral pediatric hospital, a 36-hour meropenem ASO option was implemented in the EMR for patients with sepsis requiring empiric antibiotics active against ESBL-producing organisms. We sought to evaluate the impact this initiative had on meropenem use and safety outcomes.

**Methods:**

A 36-hour ASO for meropenem was implemented on October 12^th^, 2022. We conducted a single-center, retrospective pre/post evaluation of order set implementation of all patients admitted to Riley Hospital for Children and treated with meropenem between 9/01/2019–9/01/2021 (pre-intervention) and 10/13/2022–10/13/2023 (post-intervention). The primary outcome was meropenem utilization, as measured by the number of meropenem days and doses per admission. Secondary outcomes included total hospital and ICU mean length of stay (LOS), mortality within 30 days of meropenem exposure, and 30-day readmission rate.

**Results:**

309 admissions during which meropenem was administered were included. Demographics between pre-ASO (147 patients; n = 193 admissions) and post-ASO (88 patients; n = 116 admissions) groups were similar. There was no difference in the number of meropenem days (pre and post-ASO: median = 4; p = 0.88) or the number of meropenem doses (pre-ASO: 11 vs. post-ASO: 10; p = 0.63). Secondary safety outcomes including death within 30 days of meropenem administration (8.7% vs. 8.7%; p = 0.99), hospital LOS (18 vs 15.5 days; p = 0.42), ICU admission, and 30-day readmission rate were not significantly different. The new ASO was utilized in 23.3% of admissions in the post-implementation period.

**Conclusion:**

Use of the new ASO was not widely adopted, making evaluation of impact difficult. No significant difference in meropenem use nor clinical outcomes were identified after implementation, which has informed the next Plan-Do-Study-Act (PDSA) cycle for quality improvement at our center, likely with a focus on EMR modifications and education.

**Disclosures:**

**Jack G. Schneider, MD**, MiraVista Diagnostics: Advisor/Consultant

